# Diversity in Grain Amaranths and Relatives Distinguished by Genotyping by Sequencing (GBS)

**DOI:** 10.3389/fpls.2017.01960

**Published:** 2017-11-17

**Authors:** Xingbo Wu, Matthew W. Blair

**Affiliations:** Department of Agricultural and Environmental Sciences, Tennessee State University, Nashville, TN, United States

**Keywords:** single nucleotide polymorphism (SNP) discovery, genetic diversity, genotyping by sequencing (GBS), domestication of *Amaranthus*, population structure

## Abstract

The genotyping by sequencing (GBS) method has become a molecular marker technology of choice for many crop plants because of its simultaneous discovery and evaluation of a large number of single nucleotide polymorphisms (SNPs) and utility for germplasm characterization. Genome representation and complexity reduction are the basis for GBS fingerprinting and can vary by species based on genome size and other sequence characteristics. Grain amaranths are a set of three species that were domesticated in the New World to be high protein, pseudo-cereal grain crops. The goal of this research was to employ the GBS technique for diversity evaluation in grain amaranth accessions and close relatives from six *Amaranthus* species and determine genetic differences and similarities between groupings. A total of 10,668 SNPs were discovered in 94 amaranth accessions with *Ape*KI complexity reduction and 10X genome coverage Illumina sequencing. The majority of the SNPs were species specific with 4,568 and 3,082 for the two grain amaranths originating in Central America *Amaranthus cruentus and A. hypochondriacus* and 3,284 found amongst both *A. caudatus*, originally domesticated in South America, and its close relative, *A. quitensis*. The distance matrix based on shared alleles provided information on the close relationships of the two cultivated Central American species with each other and of the wild and cultivated South American species with each other, as distinguished from the outgroup with two wild species, *A. powellii* and *A. retroflexus*. The GBS data also distinguished admixture between each pair of species and the geographical origins and seed colors of the accessions. The SNPs we discovered here can be used for marker development for future amaranth study.

## Introduction

Grain amaranth is an multi-species ancient pseudo-cereal crop domesticated in several regions of Latin America (Guzmán-Maldonado and Paredes-Lopez, 1998) that has mostly been lost to present day cultivation (Das, 2016). The grain amaranths include the *Amaranthus caudatus, A. cruentus*, and *A. hypochondriacus*, all developed by New World farmers but in different regions. The first of these species was domesticated in the Andes of South America while the second two species were from the Mesoamerica region of Central America and Mexico (Stetter et al., 2016). While very important to Aztec, Maya, and Inca civilization, the three species are relegated to minor crops in most of their native homelands due to crop substitution and suppression by Spanish colonization (Amicarelli and Camaggio, 2012). Despite this, grain amaranths have made resurgence in some parts of Mexico; were the object of a Rodale Institute funded breeding program in the United States; and have spread recently to Africa, with the crop growing in popularity in Kenya, Tanzania, and Uganda (Adeniji and Aloyce, 2013). For Asia, production of grain amaranths is significant in India and Nepal (Das, 2016) with increased production in China (Cheng, 2012). The loss of grain amaranths in its center of diversity makes it more difficult to study the genetic diversity of the species than crops such as maize and beans that are still widely grown in Latin America (Blair et al., 2006; Bedoya et al., 2017).

High drought and heat tolerance make amaranth a key food crop to study for the future. Amaranths are adapted to hot temperatures and are efficient in their water use (Omamt et al., 2006). They are one of the few genera among dicotyledonous plants to have developed the more efficient C4 photosynthesis method of CO_2_ fixation as compared to C3 photosynthesis typical in most other families (Sage, 2004). Grain amaranths are known to be tolerant of abiotic stresses, including water scarcity (Brenner et al., 2010), saline or poor fertility soils (Nasir et al., 2016; Saucedo et al., 2017), intense solar radiation and even severe defoliation (Castrillón-Arbeláez et al., 2012; Vargas-Ortiz et al., 2013; Jin et al., 2016). They also have few diseases although they do suffer from insect herbivory (Castrillón-Arbeláez et al., 2012; Massange-Sanchez et al., 2015).

Apart from its stress tolerance, fast growth and agronomic production, grain amaranths have many nutritional advantages including high levels of protein and a good balance of amino acids (Rastogi and Shukla, 2013). In addition, leaves of young plants can be used as a vegetable (Shukla et al., 2010) and pigment production (Teng et al., 2015). Grain amaranth seed is especially rich in lysine which is deficient in maize (De Ron et al., 2017). They are also relatively high in the cysteine and methionine which are deficient in beans and other legumes (Caselato-Sousa and Amaya-Farfán, 2012). These traits make amaranth a unique grain species compared to both cereals and pulses. Breeding programs established for amaranth have just begun and need further assistance for increasing yield (Brenner et al., 2010; Alemayehu et al., 2015; Stetter et al., 2016). Grain amaranth improvement must take into account that the grain producing species are monoecious and have a high degree of outcrossing, but can also be self-pollinated (Das, 2016).

Molecular markers have been developed and used for the major and most common crop species to date (Jiang, 2015). Genotyping with molecular markers is useful for germplasm evaluation and conservation, core-collection characterization and in breeding applications such as marker-assisted selection (MAS) (He et al., 2014). The grain amaranth have had various types of molecular markers applied to them including random amplified polymorphic DNAs (RAPDs) (Transue et al., 1994), isozymes (Chan and Sun, 1997), amplified fragment length polymorphisms (AFLPs) (Xu and Sun, 2001), and restriction fragment length polymorphisms (RFLPs) (Park et al., 2014). One program has developed 179 simple sequence repeated markers (SSRs) for amaranth but only 37 of these were evaluated in an F_2_ mapping population (Mallory et al., 2008). Meanwhile, 11 high polymorphism SSRs have been selected for phylogenetic analysis between weedy and grain amaranth (Kietlinski et al., 2014), but are limited in value due to lack of sequence data. As a way to overcome the lack of sequence information, Maughan et al. (2009) identified 27,658 single nucleotide polymorphisms (SNPs) from four diverse amaranth accessions. However, only 450 of these SNPs were subsequently validated for genetic linkage mapping by using competitive allele specific PCR (a.k.a KASP) technology (Maughan et al., 2011). While the amaranth marker studies have been useful for evolutionary and phylogenetic further germplasm characterization and marker validation is needed.

In this regard, genotyping by sequencing (GBS) is a practical, inexpensive and high throughput SNP fingerprinting method for reduced representation genome library sequencing and SNP discovery (Elshire et al., 2011). The GBS approach uses next generation sequencing (NGS) technologies for multiplex sequencing of restriction site-associated DNA. GBS has never been applied to grain amaranth as the reference whole genome sequence of the species has not become available until recently (Clouse et al., 2016; Lightfoot et al., 2017). Given its utility in detecting large numbers of SNP loci and rapidly genotyping diverse accession, we believe the GBS technique is a valuable technique for breeding of amaranth.

The goals of this research, therefore, were to apply GBS technology (1) to the study of amaranth diversity, (2) to discover SNPs for amaranth, and (3) to investigate the population structure of the grain amaranth compared to an outgroup of *Amaranthus* species in a part of the core collection from the USDA Genebank for amaranths. In this study, we benefitted greatly from a very high quality reference genome recently made available for *A. hypochondriacus* (Lightfoot et al., 2017). This sequence was based on PacBio single-molecule sequencing, Illumina high throughput reads and Hi-C-based proximity-guided assembly of the *n* = 16 haploid chromosomal complement of amaranth genomes which provided a valuable anchor to all the SNP loci and allele sequences discovered here.

## Materials and Methods

### Plant Material

A total of 95 germplasm entries of *Amaranthus* species were used in this study. The grain amaranths consisted in 75 accessions from the cultivated species in roughly similar numbers, 23 from *A. caudatus*, 28 from *A. cruentus*, and 24 from *A. hypochondriacus* (Supplementary Table S1). In terms of geographical representation, 15 genotypes were from Mexico, 13 from Peru, 15 from India, 7 from the United States, 6 from Guatemala, 3 from Bolivia, 2 from Zambia, 2 from Ecuador, and one each from Afghanistan, Argentina, Benin China, Maldives, Nigeria, Pakistan, Russia, Rwanda, Sudan, Uganda, and Zimbabwe. Therefore, the total from each continent were 8 genotypes from Africa, 19 from Asia and 47 from the America.

A group of 20 wild amaranth accessions were included and involved four additional species with one genotype of *A. palmeri*, three genotypes of *A. powellii*, 14 genotypes of *A. quitensis* and 2 genotypes of *A. retroflexus*. While *A. quitensis* is considered as a closely-related, wild ancestor of *A. caudatus*, the three other species, *A. palmeri, A. powellii*, and *A. retroflexus* are all weed amaranths that can be considered outgroup species to the grain amaranths and their close relatives. *A. quitensis* accessions are from the Andes of South America while the *A. palmeri, A powellii*, and *A. retroflexus* were collected in North America as weeds. All the genotypes were obtained from D. Brenner the curator for amaranths at the Genebank at the Central Plains Germplasm repository, United States Department of Agriculture (USDA) held at Ames, Iowa. Data was also downloaded from the Germplasm Resources Information Network (GRIN) database on the geographic origin and seed characteristics of each accession. For all grain amaranths, germplasm was selected from the USDA core collection of *Amaranthus*.

### DNA Extraction and Genotyping by Sequencing (GBS)

High molecular weight DNA that was also of high quality and purity was extracted for the GBS method using tissue from aseptically grown seedlings. To obtain fresh tissue for the extraction, seeds were sterilized in 5% HgCl_2_ for 5 min followed by 5 min rinses in autoclaved water (Bennici et al., 1992). Sterilized seed was then transferred into individual magenta box (Sigma–Alrich Co. LLC), one per genotype containing 100 ml of Murashige-Skoog media with 2 g of sucrose and placed in a growth chamber for 14 days. At that point, the whole seedlings were harvested for DNA extraction with a FastDNA^®^ kit (MP Biomedicals). Quantity and quality verification for DNA was made with a FLUOstar Omega spectrometer (BMG LABTECH) with settings at 260/280 mm absorbance ratio. A threshold value of 1.8 was used for high quality DNA determination followed by electrophoresis on 1% agarose gel for confirmation. A lyophilized aliquot of 1.5 ug DNA was prepared for each accession and GBS genotyping was then carried out for the full set of 95 amaranth accessions.

Genotyping by sequencing library and barcoding methods were done according to Elshire et al. (2011). Briefly, the genomic DNA was digested with the restriction enzyme (*Ape*KI) followed by ligation with a barcode adaptor and a common Illumina sequencing adaptor. Given the small size of the *Amaranthus* genome (∼500 Mb), we used the restriction enzyme indicated for GBS genome complexity reduction that has been used in rice (De Leon et al., 2016), another small genome species. This same enzyme has functioned well for GBS in maize (Ertiro et al., 2015) and soybean (Heim and Gillman, 2017) with larger genomes. Size selection using magnetic beads after digestion and ligation with adapters was for DNA fragments of approximately 300 bp (Rohland and Reich, 2012). Single-end sequencing of the 95-plex library was performed with the single-lane sequencer Illumina HiSeq 2000 (Illumina Inc. San Diego, CA, United States) at the Institute of Biotechnology of Cornell University.

### Data Analysis, SNP Identification, and Evaluation

The raw sequence data was analyzed with the GBS discovery pipeline in TASSEL software (Glaubitz et al., 2014). The FASTQ raw files and sample key files, with information of plate layout and bar codes for each genotype, were used to construct a GBS database for the identification of SNPs. Only the sequence reads containing bar code sequence followed by the sticky end sequence of an *Ape*KI restriction enzyme cut site (CWGC) were trimmed to 64 bases and stored in the European Bioinformatics Institute (EBI) database (accession number pending).

Reads that had no matching barcode or cut site remnant were excluded from the analysis, as well as reads containing unidentified bases (N) and reads with adapter dimers. Subsequently, the bar-coded sequence reads with tags present more than three times were sorted and collapsed into unique sequence tags with position information, and then aligned with the DOE-JGI sponsored database for the *Amaranthus hypochondriacus* genome v2.1 as described in Lightfoot et al. (2017) and found at http://phytozome.jgi.doe.gov/. The Burrows-Wheeler Aligner (BWA) algorithm (Li and Durbin, 2009) was used for genome alignment to that assembly. As a setting in this software, only the tags with a perfect match to reference genome were called for SNP discovery. We also used a no-references genome approach, using UNEAK subprogram in TASSEL; however, we get fewer useful SNPs than if we use the reference genome. All newly-discovered SNPs were scored for coverage, depth and genotypic information. The quality score of 10 was applied for the validation of any given locus. Average unique SNP frequency was calculated per accession in each species group due to the uneven number of accessions composing each species group.

### Population Structure and Genetic Diversity

Population structure was done in two steps. First, all the species were analyzed together and then only the grain amaranth species (*A. caudatus, A. cruentus*, and *A. hypochondriacus*), plus *A. quitensis* as a closely related wild species to the first of these grain amaranths, were analyzed separately. Structure was inferred for both groups by using model-based Bayesian framework for variation encoded in fastStructure software (Raj et al., 2014). fastStructure was preferrable given its capacity to deal with large number of SNP loci and variants in a study with multiple species such as the germplasm set from the *Amaranthus* genus evaluated here.

Subpopulations (*K*) ranged from *K* = 2–10. The python script ChooseK.py included in the fastStructure package was used to choose the number of subpopulations that maximized the marginal likelihood of the number of populations found. A Q matrix was visualized for the same grain and wild relative amaranths by DISTRUCT v1.1 to represent genome representativeness of the different genotypes belonging to each species by different color coding (Rosenberg, 2004). A distance matrix generated with TASSEL software was used for principal coordinate analysis (PCoA) of the same four species using the program XLSTAT v2017.02 made for Windows (Addinsoft XLSTAT, 2013). The two most distant species, *A. powellii* and *A. retroflexus*, were not used in the second step of population structure and in the principal component analyses as species relationships and accession classification were of most interest for the grain amaranths themselves.

However, so as to root the grain amaranths with weedy *Amaranthus* species, a phylogenetic tree was drawn for all the analyzed species, including the outgroup representatives of wild *A. powellii* and wild *A. retroflexus*, the dendrogram was based on UPGMA option in MEGA 7 (Kumar et al., 2016), with 500 bootstraps for nodal probability estimates. Subfigures were drawn based on the same phylogentic tree, but showing geographic origin and seed color of the accessions using Powerpoint software from Microsoft Office^®^. Finally, a Venn diagram was used to visualize the SNP loci shared among the six *Amaranthus* species. A diagram of overlapping SNP loci was generated using the online program InteractiVenn (Heberle et al., 2015). The genetic diversity and population structure of the six *Amaranthus* species were further investigated by analysis of molecular variance (AMOVA) by Arlequin 3.5 (Excoffier and Lischer, 2010).

## Results

### Genome-Wide Discovery of SNPs

Sequencing of the *Ape*KI genomic complexity-reduction libraries generated 24.8 Gb of raw sequence data, consisting of 510,408,206 raw sequence reads from 95 wild and grain amaranth accessions. Of these reads, a total of 279,025,903 (54.7%) contained good barcode sequences allowing them to be unequivocally arranged as non-chimeric amaranth genome sequences. In the next step, 364,377 sequence tags were analyzed for genome-wide SNP discovery, of which 233,309 (64.0%) were successfully aligned to the reference *Amaranthus hypochondriacus* genome in Phytozome. A total of 85,363 unique pre-filtration SNP loci were discovered based on the aligned tags against the reference genome. Among amaranth accessions, PI633586, which is an *Amaranthus palmeri* genotype, was removed because of a high level of missing data. Therefore, a total of 10,668 filtered SNPs were identified without missing data in the 94 amaranth accessions by setting read depth at ≥10 and by removing any SNP locus with minor allele frequency that was less than 5%.

Of these newly-discovered, grain and wild amaranth SNPs, 99.2% (10,587) were located on the major sequence contigs of the genome while only 0.8% (81) were located on minor contigs that have not been annotated (**Table 1**). Most of the SNPs identified in this study were A/G or C/T transition mutations (61.9%) with the most observed substitution type being C/T (31.4%). Transversion-type SNPs, including A/C, A/T, C/G, and G/T conversions, represented the other 38.1% of total SNPs discovered. The least common substitution type was the C/G transversion (6.9%), while substitutions involving A or T bases were more common. Among the identifiable SNPs, almost all were distributed in the largest sequence macromolecules representing the *n* = 16 chromosomal scaffolds of the *A. hypochondriacus* genome (**Supplementary Figure S1**). The number of filtered SNPs distributed in each scaffold varied from 1,113 (Scaffold 1) to 328 (Scaffold 16), while only 81 SNPs were distributed in minor contigs that were not associated with any of the predicted *Amaranthus* chromosomes.

**Table 1:** Numbers and frequency of unfiltered, filtered and polymorphic SNP loci generated by genotyping by sequencing (GBS) and detected by comparison of 94 different Amaranth accessions from six species (*Amaranthus caudatus, A. cruentus, A. hypochondriacus, A. powellii, A. quitensis*, and *A. retroflexus*).

		Number of SNPs		Frequency (kb of SNPs)	
Chromosome	Size (Mb)	Pre-filtration ^a^	Filtered ^b^	Pre-filtration ^a^	Filtered ^b^
1	38.1	7,835	1,113	20.6	2.9
2	35.7	7,469	993	20.9	2.8
3	30.2	5,807	790	19.2	2.6
4	28.3	5,834	795	20.6	2.8
5	25.7	4,509	662	17.6	2.6
6	24.6	5,181	681	21.0	2.8
7	24.4	4,540	436	18.6	1.8
8	23.8	5,174	690	21.8	2.9
9	22.7	4,707	668	20.7	2.9
10	22.7	8,325	698	36.7	3.1
11	22.3	4,586	643	20.6	2.9
12	22.1	4,820	624	21.9	2.8
13	20.7	4,429	510	21.4	2.5
14	20.2	4,174	515	20.7	2.6
15	17.5	3,744	441	21.4	2.5
16	17.0	2,775	328	16.4	1.9
Subtotal	395.8	83,909	10,587	340.0	42.4

*^a^ Number and frequency of SNPs physically mapped in *Amaranthus hypochondriacus* genome. ^b^ Number and frequency of SNPs retained after the filtration process. The SNPs were located to the longest sequence contigs representing the haploid number of chromosomes (*n* = 16) from the whole genome sequencing of *A. hypochondriacus*.*

### Population Structure and Genetic Relationships of *Amaranthus*

**FIGURE 1:**
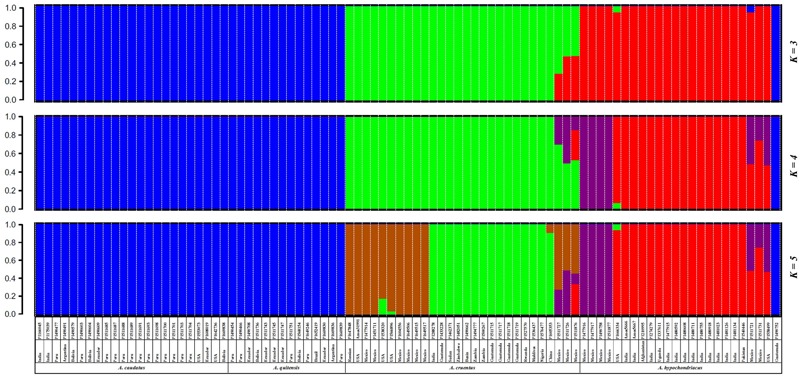
Population structure for three grain amaranth species (*Amaranthus caudatus, A. cruentus, A. hypochondriacus*) and one relative (*A. quitensis*) showing a clustering bar chart based on different population numbers (*K* = 3–5) generated by a Bayesian model and no prior classification. Plant introduction (PI) origin, accession number and species information is presented below each bar. Germplasm selected from the USDA core collection of *Amaranthus*.

All SNPs with MAF ≥ 0.05 were used to infer the genetic relationships and population structure of the amaranth accessions in this study. Population structure analysis indicated that the likely range of *K*-values was from 3 to 5 for the full group of six species and 94 accessions evaluated with the GBS method (**Supplementary Figure S2**). However, given that the small number of genotypes of *A. powellii* and *A. retroflexus* had very little shared ancestry with the other species we did a primary population structure analysis for the numerically larger group of more closely related genotypes exclusively from the species *A. caudatus, A. cruentus, A. hypochondriacus*, and *A. quitensis*, which were of primary interest (**Figure 1**).

In this part of the study concentrating on the four species just mentioned, we found a likely range of *K*-values of 3–5 for these grain amaranths as shown in the three consecutive bar diagrams in **Figure 1** and described below. The Bayesian-based structure analysis at *K* = 3 clustered the amaranth accessions mainly according to their species but combined *A. caudatus* and *A. quitensis* together as one group, and clustered *A. hypochondriacus* and *A. cruentus* as two separate groups with some admixture in a group of genotypes exclusively from Mexico. As the value of *K* increased, *A. hypochondriacus* group and *A. cruentus* group separated into two subgroups based on the geographic origins of the accession within them and seven admixed *A. cruentus/A. hypochondriacus* genotypes appeared as a new grouping.

At *K* = 4, an intermediate group of admixed and potentially hybrid *A. cruentus* × *A. hypochondriacus* genotypes was found. These seven admixed accessions (now in purple) appeared on the edge of the species division between structure groupings for *A. hypochondriacus* and *A. cruentus* and were all from Mexico. At *K* = 5, a group of 10 *A. cruentus* genotypes (now in brown) separated from other accessions within the species, and were unique in being from Mexico, Russia, and the United States, as compared to all remaining *A. cruentus* without admixture which were from Guatemala and a range of African and Asian countries. Interestingly at *K* = 5, *A. caudatus* and *A. quitensis* group didn’t show any separation either by their species assignment or by geographic origin, with most coming from Argentina, Bolivia, Ecuador, and Peru except for one Indian accession (PI166045).

**FIGURE 2:**
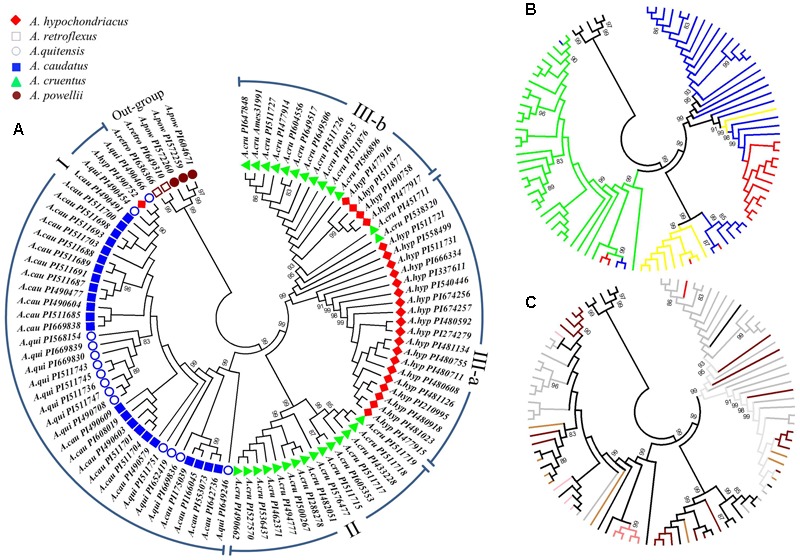
Phylogenetic dendrogram of 94 accessions from six amaranth species (*Amaranthus caudatus, A. cruentus, A. hypochondriacus, A. powellii, A. quitensis* and *A. retroflexus*). **(A)** Taxonomic classification and color coding based on populations structure analysis and plant introduction (PI) entries (clusters representing groups and subgroups are indicated in Roman numerals around the perimeter of this subfigure). **(B)** Geographic origin of each accession (color coded as green = from South/Central America, red = from Asia, blue = from North America, yellow = from Africa, black = belonging to the outgroup of weedy species). **(C)** The seed color of each accession as downloaded from the USDA Germplasm Resource Information Network (color coded as black line = black seed, brown line = brown seed, dark brown line = dark brown seed, gray line = white or cream seed, pink line = pink seed, red line = red seed, tan line = light tan seed). Germplasm selected from the USDA core collection of *Amaranthus*.

**FIGURE 3:**
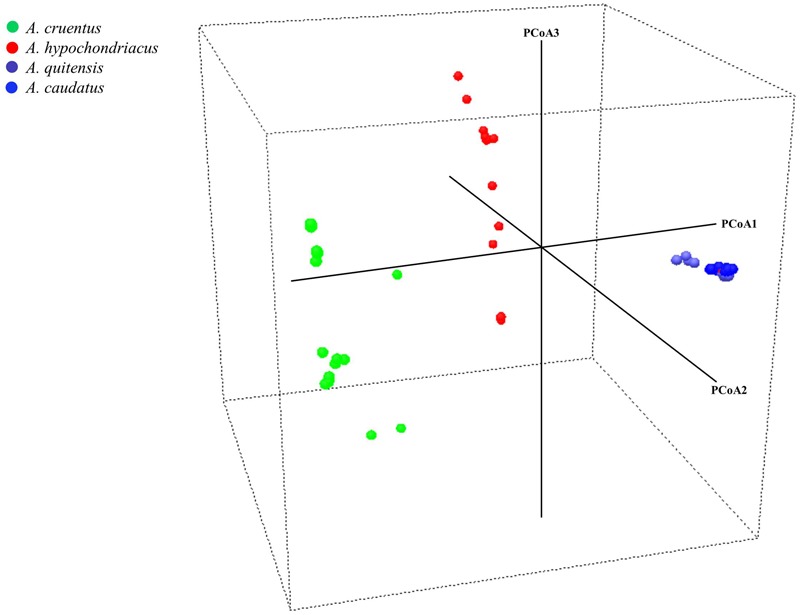
Principal coordinate analysis for three grain amaranth species (*Amaranthus caudatus, A. cruentus, A. hypochondriacus*) and one relative (*A. quitensis*). Each dot represents an accession and the color coding is based on species identification from USDA Germplasm Resources Information Network. Germplasm selected from the USDA core collection of *Amaranthus*.

A UPGMA phylogenetic tree based on the 10,668 SNPs classified the 94 accessions into three major clusters (**Figure 2**). These included: cluster I with 38 accessions that contained all the *A. caudatus* species accessions along with one accession (PI490752) mistakenly registered as *A. hypochondriacus*, plus all the closely related wild accessions belonging to the species *A. quitensis*. Cluster II was composed of 15 accessions that were all from the *A. cruentus* species. Cluster III had 36 accessions that were subdivided into two sub-clusters, with sub-cluster (a) composed of accessions from *A. hypochondriacus* and accessions from *A. cruentus* and sub-cluster (b) composed of 4 accessions from *A. hypochondriacus* and 11 accessions from *A. cruentus*. Meanwhile, all representatives of the two outgroup species, *A. retroflexus* and *A. powellii*, used in the complete analysis and consisting of five of genotypes of weedy amaranths were clustered separately from all other groups. Bootstrap values for these divisions were all very high at 99% probability values showing the high reliability of the phylogeny based on so many markers and the PCoA (**Figure 3**) validated the UPGMA findings.

Geographic origins of the accessions and seed color of each genotype are shown in the sub-figures of **Figure 2**. The first of these sub-figures shows that almost all of the *A. caudatus* and *A. quitensis* accessions were from South America except for PI166045 and PI175039 from India, which were clustered at bootstrap value of 99% with PI553073 and PI642736 from the United States (**Figure 2B**). This grouping may reflect adaptation to hotter, non-highland environments, since growing conditions in India and the United States would be different from those of highland Argentina, Bolivia, Ecuador, and Peru mostly in terms of temperature and probably photoperiod. The distinctiveness of 8 African accessions of *A. cruentus* and 11 Asian accessions of *A. hypochondriacus*, within their respective species and the sub-clusters mentioned above, is also shown in this sub-figure. Meanwhile, the seed color distinctions are evident within these two groups, both having darker colors of light to dark brown seeds, reflective of their dual purpose function as grain and vegetable amaranths. The seed color distinctions in the South American amaranths showed that *A. quitensis*, grouped at bootstrap values of 83%, have either black or dark brown seed while *A. caudatus*, in various subgroupings, can be separated by having white/cream, light tan, pink and red colored seed (**Figure 2C**).

In the PCoA (**Figure 3**), the main axis of PCoA1 separated South American originating species from North American originating ones with *A. caudatus* and *A. quitensis* clustered tightly together showing little dispersal from a centroid for both species and therefore little diversity either between these two species or among them.

Meanwhile, PCoA2 and PCoA3 axes separated *A. cruentus* and *A. hypochondriacus* and showed high dispersal and high diversity within each species and across the two species when considered together, showing that some overlap may explain the inter-specific admixtures seen in the population structure described above.

To investigate the SNP loci distribution in the different species involved in this study, the 94 accessions were assigned into six groups according to their species and number of unique validated SNPs in each group was determined with the criteria of sequence reading depth ≥10 and minor allele frequency (MAF) ≥ 0.05 (**Table 2**). Large variation was observed for total SNP and unique allele numbers in each group. The highest total SNP number was in the *A. cruentus* group having 5,953 loci with 4,568 being unique. This was followed by *A. hypochondriacus* group with 4,406 SNPs and 3,082 being unique, the *A. quitensis* group with 3,754 SNPs and 1,338 being unique, *A. caudatus* group with 3,693 SNPs and 1,344 being unique. The wild species *A. powellii* group had 3,322 SNPs with 2,513 unique and *A. retroflexus* group had 1,092 SNPs with 610 unique. Since the number of accession per groups varied, the average unique allele number per accession in each species group was calculated to compare the diverse of each species in this study. As an outgroup, *A. powellii* and *A. retroflexus* group had the highest average number of unique alleles (1,282 SNPs per accession) with total of 3,847 unique alleles in three accessions, while *A. caudatus* group had the lowest average unique alleles (110 SNPs per accession) with 2,521 unique alleles in 23 accessions.

**Table 2:** Distribution of unique SNP loci in six amaranth species (*Amaranthus caudatus, A. cruentus, A. hypochondriacus, A. powellii, A. quitensis*, and *A. retroflexus*).

Taxonomy groups	Species	Total accessions	Total SNPs	Unique SNPs^∗^	Average unique SNPs
*A. powellii*	Outgroup	3	3,322	2,513	838
*A. retroflexus*	Outgroup	2	1,092	610	305
*A. caudatus*	Grain Amaranth	23	3,693	1,344	58
*A. cruentus*	Grain Amaranth	28	5,953	4,568	163
*A. hypochondriacus*	Grain Amaranth	24	4,406	3,082	128
*A. quitensis*	Wild Amaranth	14	3,754	1,338	96

*^∗^SNPs were not shared between groups.*

**FIGURE 4:**
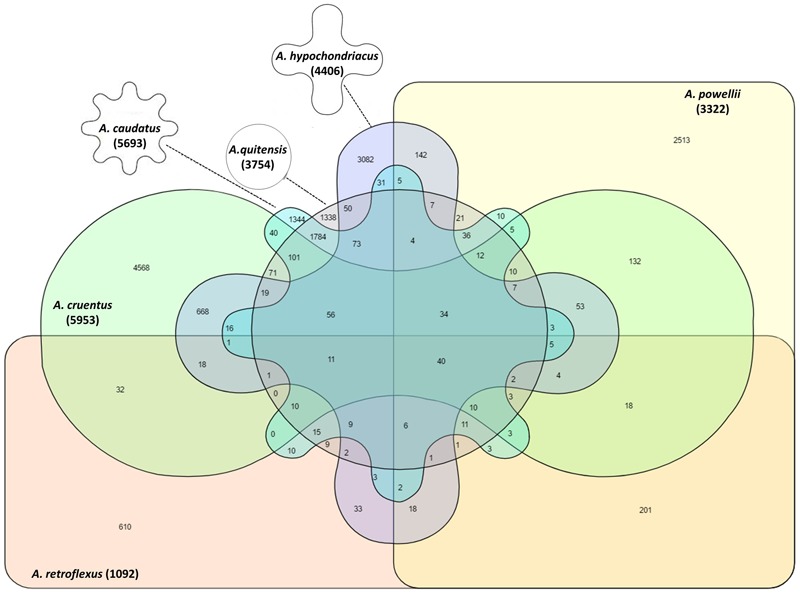
Venn diagram to show the presence, average and overlap of SNPs in six different amaranth species. Number in parenthesis below each species name indicates total SNP number within that species. Germplasm selected from the USDA core collection of *Amaranthus*.

The pattern of shared alleles showed large variation within six *Amaranthus* species (**Figure 4**). The *A. caudatus* and *A. quitensis* group had the most shared alleles (1,338) compared to the alleles shared between other species groups. No shared allele was observed between *A. caudatus, A. cruentus*, and *A. retroflexus* groups, as well as in *A. cruentus, A. quitensis*, and *A. retroflexus* groups. There were more shared SNPs between the three grain amaranths and *A. quitensis* than between these and *A. powellii* or *A. retroflexus*. There were only 40 SNPs shared by all of the six species groups.

Similarly, variation within the six *Amaranthus* species was supported by AMOVA analysis (**Table 3**). AMOVA analyses of six *Amaranthus* species indicated that the majority of the variance occurred among groups, in other words among the species, and accounted for 83.7% of the total variation, whereas 9.1% and 7.2% of the variation was attributed to differences within individuals and between groups, respectively.

**Table 3:** Analyses of molecular variance (AMOVAs) based on six, four and combinations of two species (*Amaranthus caudatus, A. cruentus, A. hypochondriacus, A. powellii, A. quitensis*, and *A. retroflexus*).

Source of variation	*df*	Sum of squares	Variance components	Percentage of variation
**Variation partition among six species groups**				

Among species	5	321,707.202	2,220.57627	83.7
Among individuals within species	88	59,164.312	240.76816	9.1
Within individuals	94	17,933.828	190.7854	7.2
Total	187	398,805.341	2,652.12983	

**Variation partition among grain amaranths^‡^ and *Am aranthus quitensis***				

Among species	3	405,975.259	3,073.5281	83.1
Among individuals within species	85	82,000.513	339.37332	9.2
Within individuals	89	25,450.909	285.96527	7.7
Total	177	513,426.681	3,698.86669	

**Variation partition among *Amaranthus caudatus*^∗^ and *A. quitensis***				

Among species	1	6,326.664	144.46717	14.8
Among individuals within species	35	45,417.553	465.92302	47.7
Within individuals	37	13,534.539	365.79834	37.5
Total	73	65,278.756	976.18853	

**Variation partition among *Amaranthus cruentus* and *A. hypochondriacus*^∗^**				

Among species	1	-5,267.138	-105.05664	NS^∗∗^
Among individuals within species	49	1,923.359	-76.02042	NS^∗∗^
Within individuals	51	9,755.947	191.29307	100
Total	101	6,412.168	10.21601	

*^∗^PI490752 was not included. ^∗∗^Not significant. ^‡^*Amaranthus caudatus, A. cruentus, A. hypochondriacus*, and *A. quitensis.**

Additional AMOVA analysis with three grain amaranth species (*A. caudatus, A. cruentus*, and *A. hypochondriacus*) and *A. quitensis* explained 83.1% of the variation between the four groups while a contrast of just two relative *A. caudatus* and *A. quitensis* found 37.5% within individuals, 47.7% among individuals within species and only 14.8% between species. In comparison, a similar contrast but this time only with *A. cruentus* and *A. hypochondriacus* found species differences to be non-significant and 100% of the variance to be within individuals.

## Discussion

In technical terms, GBS was shown to be an efficient method of high-throughput genotyping for amaranth. As in previous studies, GBS is bioinformatically intensive but has the ability of discovering many SNPs in a collection of diverse genotype (Elshire et al., 2011). The successful implementation of GBS, however, require proper library construction and sequence analyses, and benefit from a good reference genome for analysis. For these reasons, GBS has been most widely used for plant research programs in the most important crops like wheat (Lin et al., 2015), rice (Spindel et al., 2013), maize (Romay et al., 2013), and soybean (Heim and Gillman, 2017). In this study, we applied GBS to a group of pseudocereals and orphan crop jointly termed the grain amaranths whose reference genome has only recently been prepared (Clouse et al., 2016; Lightfoot et al., 2017).

In total, we discovered 10,668 SNPs by using a single enzyme (*Ape*KI) in the library preparation step combined with deep sequencing and comparison to the *A. hypochondriacus* reference genome (Clouse et al., 2016; Lightfoot et al., 2017) even after screening for missing data and quality parameters of these SNPs. The reference-free approach produced only 5,000 SNPs (data not shown) compared to the reference-based approach using the same settings of filtering for missing SNPs. We believe that since the *Amaranthus* genome is now of a very high quality and full length macromolecules are available that the reference genome approach is best even across related species. The number of SNP loci we discovered compared favorably with previous GBS studies that used two-enzyme digestion library preparation or both reference based and non-reference based (*de novo*) assembly methods (Stetter and Schmid, 2017; Stetter et al., 2017).

Our SNPs were developed from 94 diverse *Amaranthus* genotypes and possessed higher polymorphism compared to the 27,658 SNPs from four *A. caudatus* genotypes found by Maughan et al. (2009). Thus, our newly-discovered SNPs can be used as a complementary set to the previous SNPs for *Amaranthus* and will be valuable for SNP marker development by competitive allele specific PCR (KASP) assay which currently consists in only 450 validated markers (Maughan et al., 2011).

Genetically, the coverage of the SNPs over the reference genome showed that our GBS data can provide additional markers to complement the genetic map created from the analysis of a *A. hypochondriacus* × *A. caudatus* grain amaranth cross (Maughan et al., 2011) and can be used to improve the annotation of the reference genome by Clouse et al. (2016). For example, the large variation in SNP numbers among the largest sequence contigs showed that the *Amaranthus* chromosomes (*n* = 16) are of different physical sizes and genetic makeup (**Supplementary Figure S1**) and these distances based on Lightfoot et al. (2017) show the confidence of GBS as a technique for full genome coverage by new markers.

The high density of SNPs from our GBS data in certain parts of the physical map may reflect the euchromatin where unique sequences would be highly frequent. Lower density of SNPs from GBS in other parts of the physical showed the location of the heterochromatic space where unique sequences are uncommon but repeat sequences are common according the high quality sequencing from Lightfoot et al. (2017). Gaps in the physical map of newly-developed SNP markers for each sequence contig seemed to reflect the locations of the centromeres (indicated with stars in **Supplementary Figure S1**) of each chromosome as found by this recent publication.

Genetic diversity analyses based on the taxonomic assignment of the accessions in our study revealed large amounts of variation in each grain amaranth species as well as their close relatives. Given the uneven numbers of accessions in each species groups, average numbers of unique SNPs were investigated.

Weedy amaranth, *A. powellii* and *A. retroflexus*, possessed more unique SNPs per accession than grain amaranth. In contrast, *A. quitensis* had less average unique SNPs per accession than *A. hypochondriacus* and *A. cruentus*, but higher than *A. caudatus*, suggesting a common origin for the weedy and the grain amaranths in the Andean and a lack of overall diversity compared to the Mesoamerica grain amaranths. Among those groups, *A. quitensis* and *A. caudatus* groups shared the highest number of shared alleles, showing the closest relationship between them compare to the relationships with other groups. There were only 40 SNPs shared among all six amaranth species.

Genotyping and population structure analyses of three cultivated amaranth, *A. caudatus, A. cruentus*, and *A. hypochondriacus*, and the three wild species, *A. quitensis, A. powellii*, and *A. retroflexus*, showed a close relationship between *A. caudatus* and its progenitor species, *A. quitensis*; but strong genetic differences between the amaranths from North America and South America, and even stronger differences between the grain amaranths and the two outgroup weedy species *A. powellii* and *A. retroflexus*. Phylogenetic analysis clustered *A. quitensis* and *A. caudatus* as cluster I while *A. hypochondriacus* and *A. cruentus* accessions clustered as two separate groups (cluster II and cluster III, respectively). *A. powellii* and *A. retroflexus* were grouped together as an outgroup. The UPGMA-based phylogeny tree was consistent with the taxonomic classification of amaranth species and with population structure analyses.

Importantly for breeding of the grain amaranths, the Mesoamerican species (those from North and Central America), such as *A. hypochondriacus* and *A. cruentus*, were grouped together and were separated from the Andean or South America amaranths, *A. caudatus* and *A. quitensis* with no evidence for admixture or hybridization. The population structure analysis corresponded with the UPGMA analysis, as the group of *A. caudatus* and *A. quitensis* corresponded to the assignment in cluster I, including the mistaken assignment of one *A. caudatus* accession as an *A. hypochondriacus* entry.

The admixture and low level of separation between *A. cruentus* and *A. hypochondriacus* groups as cluster II and cluster III, respectively, validates the hypothesis long known in Mexico that these two species are related perhaps on a continuum of environmental adaptation to different altitudes. The clustering of intermediate *A. quitensis* and *A. caudatus* and high level of shared SNPs (1,784) indicated a high degree of shared ancestry for these two species, while the intermediate level of shared SNPs (938) and admixture of *A. cruentus* and *A. hypochondriacus* shows the domestication patterns of South and North American grain amaranths.

In evolutionary terms, our data agrees with the independent evolution and then domestication hypothesis for the South American grain amaranth, *A. caudatus*, being domesticated and descended from the close wild relative, *A. quitensis* (Sauer, 1967). An alternative hypothesis could be that *A. quitensis* is a weedy derivative of *A. caudatus* with pink/red to light/dark brown colored seeds compared to cultivars with white and cream, waxy or non-waxy seed (Jimenez et al., 2013). The direction of gene flow should be studied *in situ* to determine more about the relationship of the two closely related taxa.

Meanwhile, the admixture between the Mesoamerican grain amaranths, *A. cruentus* and *A. hypochondriacus* suggested that these two species were domesticated together from either one or several closely-related wild amaranths in Mexico and that these have continued to hybridize in Mexico and to a certain extent outside this primary center of diversity. Finally, the clear separation of the grain amaranth, *A. hypochondriacus*, from the weed amaranth, *A. powellii*, also confirmed that they are not closely related to each other as previous studies concluded (Mallory et al., 2008; Stetter and Schmid, 2017) and contradict the hypothesis that *A. powellii* was the progenitor of *A. hypochondriacus* as originally proposed by Sauer (1967).

The clustering of accessions by geographic origin in the UPGMA analysis was very notable in the Mesoamerican grain amaranths. In the first of two clear examples, all the African accessions of *A. cruentus* can be found clustering together reflecting a possible founder effect for germplasm on that continent. This group would be derived from a specific set of North American germplasm that is distinct from the majority of *A. cruentus*. In the second example, a group of *A. hypochondriacus* accessions from Asia clustered together. In both cases, the selection for vegetable use might have played a role in differentiating these groups. Some Mesoamerican derived grain amaranths seem to have been selected for dual purpose use of both leaves and seed consistent with the traditions of leafy amaranths in Africa and Asia (Das, 2016).

Another example of the utility of GBS to accurately identify geographic and species groupings is found in a misidentified *A. caudatus* accession PI 490752, characterized as *A. hypochondriacus* by 11 SSR markers (Kietlinski et al., 2014), but which had consistent phylogeny and population structure results, showing it should be assigned into the *A. caudatus* group. This genotype was collected from Guatemala, which has climate similarity to regions of the Andes highlands showing that an Andean grain amaranth from the *A. caudatus* grouping could adapt to this central Americas environment. We suggest the re-analysis of PI490752 for morphological characteristics which could correct the possible misclassification as identified by the GBS markers.

In summary, this study has used the GBS method to advance amaranth science both in technical and taxonomic terms. Our finding of consistency of GBS classification geographic origin and seed color indicated that population structure must be taken into account for the evaluation of marker × trait associations. In this regard, genome wide association studies (GWAS) holds great promise in the grain amaranths but must consider the distinct South American and North American origins of the accessions studied. Furthermore, the reference genome approach of GBS had the advantage of giving genome specific SNP locations over all 16 chromosomes something that we would not be able to do with the reference-free approach since this method does not have position information.

The utility of GWAS association mapping in the grain amaranths may be greatest in understanding their high drought tolerance, associated with the accumulation of compatible solutes (Huerta-Ocampo et al., 2011), and the expression of stress-related genes and transcription factors (Huerta-Ocampo et al., 2014; Massange-Sánchez et al., 2016). In GWAS, unique SNPs can be used to distinguish genotypes with novel functional alleles for drought and heat tolerance. The high amount of diversity available in the amaranths especially those from regions of rapid climate change in North and South America (Friedman et al., 2013) can lead to the discovery of new genes for resistance/tolerance to abiotic stresses that are important to grain amaranth breeding as well as to improvement of a range of other crops.

## Author Contributions

XW carried out all experiments, prepared tables and figures and wrote the manuscript draft. MB organized and funded research in amaranth diversity through an Evans Allen Grant from Tennessee State University and edited the manuscript versions.

## Conflict of Interest Statement

The authors declare that the research was conducted in the absence of any commercial or financial relationships that could be construed as a potential conflict of interest. The reviewer MG and handling Editor declared their shared affiliation.

## Supplementary Material

FIGURE S1 Distribution of the filtered SNP loci on a physical map of the 16 largest sequence scaffolds represent the chromosome of *Amaranthus hypochondriacus* v2.1 genome from Phytozome database. Red asterisks represent the estimated centromere position of each chromosome according to Lightfoot et al. (2017).

FIGURE S2 Population structure for six amaranth species (*Amaranthus caudatus, A. cruentus, A. hypochondriacus, A. powellii, A. quitensis*, and *A. retroflexus*) showing a clustering bar chart based on three population numbers (*K* = 3–5) generated by a Bayesian model and no prior classification. Plant introduction (PI) origin, accession number and species information is presented below each bar. Germplasm selected from the USDA core collection of *Amaranthus*.

## References

[B1] Addinsoft XLSTAT (2013). *Data Analysis and Statistics Software for Microsoft Excel.* New York, NY: Addinsoft.

[B2] Adeniji O. Aloyce A. (2013). Farmers’ participatory identification of horticultural traits: developing breeding objectives for vegetable amaranth in Tanzania. *J. Crop Improv.* 27 309–318. 10.1080/15427528.2013.768318

[B3] Alemayehu F. Bendevis M. A. Jacobsen S. E. (2015). The potential for utilizing the seed crop amaranth (*Amaranthus* spp.) in East Africa as an alternative crop to support food security and climate change mitigation. *J. Agron. Crop Sci.* 201 321–329. 10.1111/jac.12108

[B4] Amicarelli V. Camaggio G. (eds). (2012). “*Amaranthus*: a crop to rediscover,” in *Forum Ware International* (Vienna: International Society of Commodity Science and Technology (IGWT)) 4–11. Available at: https://static1.squarespace.com/static/53596c97e4b095832d6a11aa/t/5507de1be4b08d28db65af12/1426578971805/Amaranthus-+A+crop+to+rediscover.pdf

[B5] Bedoya C. A. Dreisigacker S. Hearne S. Franco J. Mir C. Prasanna B. M. (2017). Genetic diversity and population structure of native maize populations in Latin America and the Caribbean. *PLOS ONE* 12:e0173488. 10.1371/journal.pone.0173488 28403177 PMC5389613

[B6] Bennici A. Schiff S. Bovelli R. (1992). *In vitro* culture of species and varieties of four *Amaranthus* L. species. *Euphytica* 62 181–186. 10.1007/bf00041752

[B7] Blair M. W. Giraldo M. C. Buendia H. F. Tovar E. Duque M. C. Beebe S. E. (2006). Microsatellite marker diversity in common bean (*Phaseolus vulgaris* L.). *Theor. Appl. Genet.* 113 100–109. 10.1007/s00122-006-0276-4 16614831

[B8] Brenner D. Baltensperger D. Kulakow P. Lehmann J. Myers R. Slabbert M. (2010). Genetic resources and breeding of *Amaranthus*. *Plant Breed. Rev.* 19 227–285. 10.3389/fpls.2016.00816 27375666 PMC4894896

[B9] Caselato-Sousa V. M. Amaya-Farfán J. (2012). State of knowledge on amaranth grain: a comprehensive review. *J. Food Sci.* 77 R93–R104. 10.1111/j.1750-3841.2012.02645.x 22515252

[B10] Castrillón-Arbeláez P. A. Martínez-Gallardo N. Arnaut H. A. Tiessen A. Délano-Frier J. P. (2012). Metabolic and enzymatic changes associated with carbon mobilization, utilization and replenishment triggered in grain amaranth (*Amaranthus cruentus*) in response to partial defoliation by mechanical injury or insect herbivory. *BMC Plant Biol.* 12:163. 10.1186/1471-2229-12-163 22966837 PMC3515461

[B11] Chan K. Sun M. (1997). Genetic diversity and relationships detected by isozyme and RAPD analysis of crop and wild species of *Amaranthus*. *Theor. Appl. Genet.* 95 865–873. 10.1007/s001220050637 24169893

[B12] Cheng J. (2012). Industrialization development of microecological food based on green plant: a case of grain amaranth [J]. *Agric. Eng.* 11 013.

[B13] Clouse J. Adhikary D. Page J. Ramaraj T. Deyholos M. Udall J. (2016). The amaranth genome: genome, transcriptome, and physical map assembly. *Plant Genome* 9 1–14. 10.3835/plantgenome2015.07.0062 27898770

[B14] Das S. (ed.) (2016). “Distribution and maintenance of amaranth germplasm worldwide,” in *Amaranthus: A Promising Crop of Future* (Berlin: Springer) 99–106. 10.1007/978-981-10-1469-7_7

[B15] De Leon T. B. Linscombe S. Subudhi P. K. (2016). Molecular dissection of seedling salinity tolerance in rice (*Oryza sativa* L.) Using a High-Density GBS-Based SNP Linkage Map. *Rice* 9 52. 10.1186/s12284-016-0125-2 27696287 PMC5045836

[B16] De Ron A. M. Sparvoli F. Pueyo J. J. Bazile D. (2017). Protein crops: food and feed for the future. *Front. Plant Sci.* 8:105. 10.3389/fpls.2017.00105 28220133 PMC5292564

[B17] Elshire R. J. Glaubitz J. C. Sun Q. Poland J. A. Kawamoto K. Buckler E. S. (2011). A robust, simple genotyping-by-sequencing (GBS) approach for high diversity species. *PLOS ONE* 6:e19379. 10.1371/journal.pone.0019379 21573248 PMC3087801

[B18] Ertiro B. T. Ogugo V. Worku M. Das B. Olsen M. Labuschagne M. (2015). Comparison of Kompetitive Allele Specific PCR (KASP) and genotyping by sequencing (GBS) for quality control analysis in maize. *BMC Genomics* 16:908. 10.1186/s12864-015-2180-2 26545737 PMC4636831

[B19] Excoffier L. Lischer H. E. (2010). Arlequin suite ver 3.5: a new series of programs to perform population genetics analyses under Linux and Windows. *Mol. Ecol. Resour.* 10 564–567. 10.1111/j.1755-0998.2010.02847.x 21565059

[B20] Friedman A. R. Hwang Y.-T. Chiang J. C. Frierson D. M. (2013). Interhemispheric temperature asymmetry over the twentieth century and in future projections. *J. Clim.* 26 5419–5433. 10.1175/JCLI-D-12-00525.1

[B21] Glaubitz J. C. Casstevens T. M. Lu F. Harriman J. Elshire R. J. Sun Q. (2014). TASSEL-GBS: a high capacity genotyping by sequencing analysis pipeline. *PLOS ONE* 9:e90346. 10.1371/journal.pone.0090346 24587335 PMC3938676

[B22] Guzmán-Maldonado S. Paredes-Lopez O. (1998). “Functional products of plants indigenous to Latin America: amaranth, quinoa, common beans, and botanicals,” in *Functional Foods: Biochemical and Processing Aspects* ed. Mazza G. (Lancaster, PA: Technomic Publishing Co. Inc.) 293–328.

[B23] He J. Zhao X. Laroche A. Lu Z. X. Liu H. Li Z. (2014). Genotyping-by-sequencing (GBS), an ultimate marker-assisted selection (MAS) tool to accelerate plant breeding. *Front. Plant Sci.* 5:484. 10.3389/fpls.2014.00484 25324846 PMC4179701

[B24] Heberle H. Meirelles G. V. da Silva F. R. Telles G. P. Minghim R. (2015). InteractiVenn: a web-based tool for the analysis of sets through Venn diagrams. *BMC Bioinformatics* 16:169. 10.1186/s12859-015-0611-3 25994840 PMC4455604

[B25] Heim C. B. Gillman J. D. (2017). Genotyping-by-sequencing-based investigation of the genetic architecture responsible for a ∼sevenfold increase in soybean seed stearic acid. *G3 (Bethesda)* 7 299–308. 10.1534/g3.116.035741 27866151 PMC5217118

[B26] Huerta-Ocampo J. A. Barrera-Pacheco A. Mendoza-Hernandez C. S. Espitia-Rangel E. Mock H.-P. Barba de la Rosa A. P. (2014). Salt stress-induced alterations in the root proteome of *Amaranthus cruentus* L. *J. Proteome Res.* 13 3607–3627. 10.1021/pr500153m 24942474

[B27] Huerta-Ocampo J. A. León-Galván M. F. Ortega-Cruz L. B. Barrera-Pacheco A. De León-Rodríguez A. Mendoza-Hernández G. (2011). Water stress induces up-regulation of DOF1 and MIF1 transcription a factors and down-regulation of proteins involved in secondary metabolism in amaranth roots (*Amaranthus hypochondriacus* L.). *Plant Biol.* 13 472–482. 10.1111/j.1438-8677.2010.00391.x 21489098

[B28] Jiang G. L. (2015). “Molecular marker-assisted breeding: a plant breeder’s review,” in *Advances in Plant Breeding Strategies: Breeding, Biotechnology and Molecular Tools* eds Al-Khayri J. M. Jain S. M. Johnson D. V. (Cham: Springer International Publishing) 431–472. 10.1007/978-3-319-22521-0_15

[B29] Jimenez F. R. Maughan P. J. Alvarez A. Kietlinski K. D. Smith S. M. Pratt D. B. (2013). Assessment of genetic diversity in Peruvian amaranth (*Amaranthus caudatus* and *A. hybridus*) germplasm using single nucleotide polymorphism markers. *Crop Sci.* 53 532–541. 10.2135/cropsci2012.07.0413

[B30] Jin H. Xu M. Chen H. Zhang S. Han X. Tang Z. (2016). Comparative proteomic analysis of differentially expressed proteins in *Amaranthus hybridus* L. Roots Under Cadmium Stress. *Water Air Soil Pollut.* 227 1–12. 10.1007/s11270-016-2914-z

[B31] Kietlinski K. D. Jimenez F. Jellen E. N. Maughan P. J. Smith S. M. Pratt D. B. (2014). Relationships between the weedy (*Amaranthaceae*) and the grain amaranths. *Crop Sci.* 54 220–228. 10.1016/j.ympev.2016.12.029 28057554

[B32] Kumar S. Stecher G. Tamura K. (2016). MEGA7: Molecular Evolutionary Genetics Analysis version 7.0 for bigger datasets. *Mol. Biol. Evol.* 33 1870–1874. 10.1093/molbev/msw054 27004904 PMC8210823

[B33] Li H. Durbin R. (2009). Fast and accurate short read alignment with Burrows–Wheeler transform. *Bioinformatics* 25 1754–1760. 10.1093/bioinformatics/btp324 19451168 PMC2705234

[B34] Lightfoot D. J. Jarvis D. E. Ramaraj T. Lee R. Jellen E. N. Maughan P. J. (2017). Single-molecule sequencing and Hi-C-based proximity-guided assembly of amaranth (*Amaranthus hypochondriacus*) chromosomes provide insights into genome evolution. *BMC Biol.* 15:74. 10.1186/s12915-017-0412-4 28854926 PMC5577786

[B35] Lin M. Cai S. Wang S. Liu S. Zhang G. Bai G. (2015). Genotyping-by-sequencing (GBS) identified SNP tightly linked to QTL for pre-harvest sprouting resistance. *Theor. Appl. Genet.* 128 1385–1395. 10.1007/s00122-015-2513-1 25851002

[B36] Mallory M. A. Hall R. V. McNabb A. R. Pratt D. B. Jellen E. N. Maughan P. J. (2008). Development and characterization of microsatellite markers for the grain amaranths. *Crop Sci.* 48 1098–1106. 10.2135/cropsci2007.08.0457

[B37] Massange-Sánchez J. A. Palmeros-Suárez P. A. Espitia-Rangel E. Rodríguez-Arévalo I. Sánchez-Segura L. Martínez-Gallardo N. A. (2016). Overexpression of grain amaranth (*Amaranthus hypochondriacus*) AhERF or AhDOF transcription factors in *Arabidopsis thaliana* increases water deficit-and salt-stress tolerance, respectively, via contrasting stress-amelioration mechanisms. *PLOS ONE* 11:e0164280. 10.1371/journal.pone.0164280 27749893 PMC5066980

[B38] Massange-Sanchez J. A. Palmeros-Suarez P. A. Martinez-Gallardo N. A. Castrillon-Arbelaez P. A. Avilés-Arnaut H. Alatorre-Cobos F. (2015). The novel and taxonomically restricted *Ah24* gene from grain amaranth (*Amaranthus hypochondriacus*) has a dual role in development and defense. *Front. Plants Sci.* 6:602. 10.3389/fpls.2015.00602 26300899 PMC4524895

[B39] Maughan P. Smith S. Fairbanks D. Jellen E. (2011). Development, characterization, and linkage mapping of single nucleotide polymorphisms in the grain amaranths (*Amaranthus* sp.). *Plant Genome* 4 92–101. 10.3835/plantgenome2010.12.0027

[B40] Maughan P. J. Yourstone S. M. Jellen E. N. Udall J. A. (2009). SNP discovery via genomic reduction, barcoding, and 454-pyrosequencing in amaranth. *Plant Genome* 2 260–270. 10.1186/1471-2164-13-724 23259499 PMC3549761

[B41] Nasir F. Islam S. Munna G. Ray S. Awal R. (2016). Effectiveness of *Amaranthus gangeticus* in arsenic extraction from soil. *J. Sci. Res.* 8 71–79. 10.3329/jsr.v8i1.24359

[B42] Omamt E. Hammes P. Robbertse P. (2006). Differences in salinity tolerance for growth and water-use efficiency in some amaranth (*Amaranthus* spp.) *genotypes*. *N. Z. J. Crop Hortic. Sci.* 34 11–22. 10.1080/01140671.2006.9514382

[B43] Park Y.-J. Nishikawa T. Matsushima K. Minami M. Nemoto K. (2014). A rapid and reliable PCR-restriction fragment length polymorphism (RFLP) marker for the identification of *Amaranthus cruentus* species. *Breed. Sci.* 64 422–426. 10.1270/jsbbs.64.422 25914599 PMC4267319

[B44] Raj A. Stephens M. Pritchard J. K. (2014). fastSTRUCTURE: variational inference of population structure in large SNP data sets. *Genetics* 197 573–589. 10.1534/genetics.114.164350 24700103 PMC4063916

[B45] Rastogi A. Shukla S. (2013). Amaranth: a new millennium crop of nutraceutical values. *Crit. Rev. Food Sci. Nutr.* 53 109–125. 10.1080/10408398.2010.517876 23072528

[B46] Rohland N. Reich D. (2012). Cost-effective, high-throughput DNA sequencing libraries for multiplexed target capture. *Genome Res.* 22 939–946. 10.1101/gr.128124.111 22267522 PMC3337438

[B47] Romay M. C. Millard M. J. Glaubitz J. C. Peiffer J. A. Swarts K. L. Casstevens T. M. (2013). Comprehensive genotyping of the USA national maize inbred seed bank. *Genome Biol.* 14:R55. 10.1186/gb-2013-14-6-r55 23759205 PMC3707059

[B48] Rosenberg N. A. (2004). DISTRUCT: a program for the graphical display of population structure. *Mol. Ecol. Notes* 4 137–138. 10.1046/j.1471-8286.2003.00566.x

[B49] Sage R. F. (2004). The evolution of C4 photosynthesis. *New Phytol.* 161 341–370. 10.1111/j.1469-8137.2004.00974.x 33873498

[B50] Saucedo A. L. Hernández-Domínguez E. E. de Luna-Valdez L. A. Guevara-García A. A. Escobedo-Moratilla A. Bojorquéz-Velázquez E. (2017). Insights on structure and function of a late embryogenesis abundant protein from *Amaranthus cruentus*: an intrinsically disordered protein involved in protection against desiccation, oxidant conditions, and osmotic stress. *Front. Plant Sci.* 8:497. 10.3389/fpls.2017.00497 28439280 PMC5384071

[B51] Sauer J. D. (1967). The grain amaranths and their relatives: a revised taxonomic and geographic survey. *Ann. Mo. Bot. Gard.* 54 103–137. 10.2307/2394998

[B52] Shukla S. Bhargava A. Chatterjee A. Pandey A. C. Mishra B. K. (2010). Diversity in phenotypic and nutritional traits in vegetable amaranth (*Amaranthus tricolor*), a nutritionally underutilised crop. *J. Sci. Food Agric.* 90 139–144. 10.1002/jsfa.3797 20355024

[B53] Spindel J. Wright M. Chen C. Cobb J. Gage J. Harrington S. (2013). Bridging the genotyping gap: using genotyping by sequencing (GBS) to add high-density SNP markers and new value to traditional bi-parental mapping and breeding populations. *Theor. Appl. Genet.* 126 2699–2716. 10.1007/s00122-013-2166-x 23918062

[B54] Stetter M.G. Müller T. Schmid K. J. (2017). Genomic and phenotypic evidence for an incomplete domestication of South American grain amaranth (*Amaranthus caudatus*). *Mol. Ecol.* 26 871–886. 10.1111/mec.1397 28019043

[B55] Stetter M. G. Schmid K. J. (2017). Analysis of phylogenetic relationships and genome size evolution of the *Amaranthus* genus using GBS indicates the ancestors of an ancient crop. *Mol. Phylogenet. Evol.* 109 80–92. 10.1016/j.ympev.2016.12.029 28057554

[B56] Stetter M. G. Zeitler L. Steinhaus A. Kroener K. Biljecki M. Schmid K. J. (2016). Crossing methods and cultivation conditions for rapid production of segregating populations in three grain amaranth species. *Front. Plant Sci.* 7:816. 10.3389/fpls.2016.00816 27375666 PMC4894896

[B57] Teng X.-L. Chen N. Xiao X.-G. (2015). Identification of a catalase-phenol oxidase in betalain biosynthesis in Red Amaranth (*Amaranthus cruentus*). *Front. Plant Sci.* 6:1228. 10.3389/fpls.2015.01228 26779247 PMC4705222

[B58] Transue D. Fairbanks D. Robison L. Andersen W. (1994). Species identification by RAPD analysis of grain amaranth genetic resources. *Crop Sci.* 34 1385–1389. 10.2135/cropsci1994.0011183X003400050044x

[B59] Vargas-Ortiz E. Espitia-Rangel E. Tiessen A. Délano-Frier J. P. (2013). Grain amaranths are defoliation tolerant crop species capable of utilizing stem and root carbohydrate reserves to sustain vegetative and reproductive growth after leaf loss. *PLOS ONE* 8:e67879. 10.1371/journal.pone.0067879 23861825 PMC3701626

[B60] Xu F. Sun M. (2001). Comparative analysis of phylogenetic relationships of grain amaranths and their wild relatives (*Amaranthus*; Amaranthaceae) using internal transcribed spacer, amplified fragment length polymorphism, and double-primer fluorescent intersimple sequence repeat markers. *Mol. Phylogenet. Evol.* 21 372–387. 10.1006/mpev.2001.1016 11741380

