# Supplementary figures and images for: Diversity in Grain Amaranths and Relatives Distinguished by Genotyping by Sequencing (GBS)
Source: Front Plant Sci. 2017 Nov 17;8:1960. doi: 10.3389/fpls.2017.01960 (PMC5698268; doi:10.3389/fpls.2017.01960)

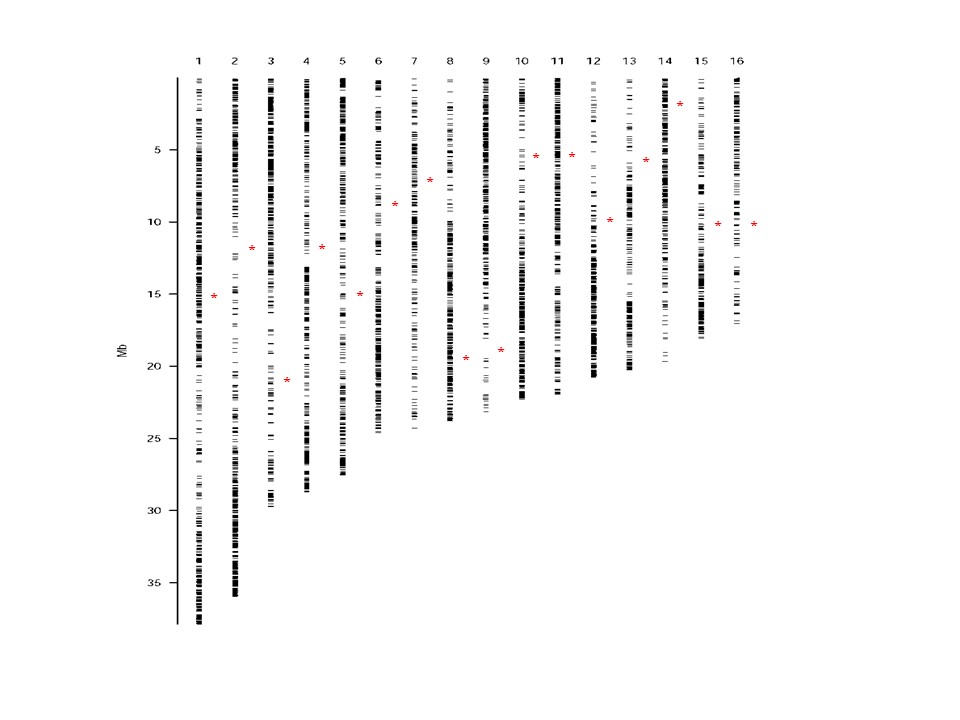

Supplement: FIGURE S1 — Distribution of the filtered SNP loci on a physical map of the 16 largest sequence scaffolds represent the chromosome of Amaranthus hypochondriacus v2.1 genome from Phytozome database. Red asterisks represent the estimated centromere position of each chromosome according to Lightfoot et al. (2017). [file Image_1.JPEG]

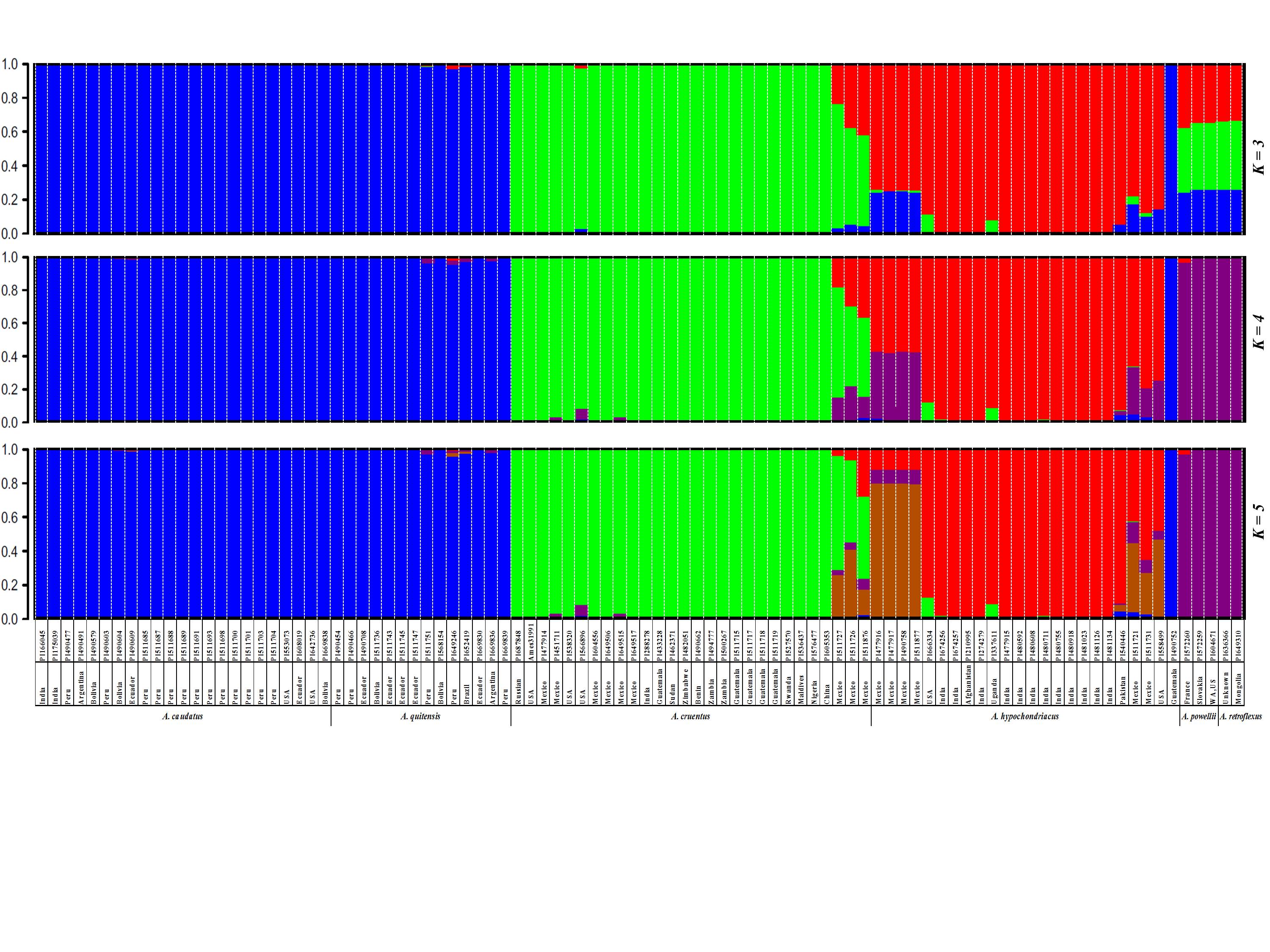

Supplement: FIGURE S2 — Population structure for six amaranth species (Amaranthus caudatus, A. cruentus, A. hypochondriacus, A. powellii, A. quitensis, and A. retroflexus) showing a clustering bar chart based on three population numbers (K = 3–5) generated by a Bayesian model and no prior classification. Plant introduction (PI) origin, accession number and species information is presented below each bar. Germplasm selected from the USDA core collection of Amaranthus. [file Image_2.JPEG]
