# Supplementary material for: Diversity in Grain Amaranths and Relatives Distinguished by Genotyping by Sequencing (GBS)
Source: Front Plant Sci. 2017 Nov 17;8:1960. doi: 10.3389/fpls.2017.01960 (PMC5698268; doi:10.3389/fpls.2017.01960)
Supplement: Supplementary file 3 [file Table_1.pdf]

Supplementary Table S1. PI number, species, origin, and seed color information of sampled amaranth accessions from the USDA core collection for the genus.

**Code**	**PI**	**Species**	**Origin**	**Seed Color**
1	PI166045	*A.caudatus*	India	Pink
2	PI175039	*A.caudatus*	India	Pink
3	PI490477	*A.caudatus*	Peru	White
4	PI490491	*A.caudatus*	Argentina	Dark Brown
5	PI490579	*A.caudatus*	Bolivia	White
6	PI490603	*A.caudatus*	Peru	Light Tan
7	PI490604	*A.caudatus*	Bolivia	White/Pink/Brown
8	PI490609	*A.caudatus*	Ecuador	Light Tan
9	PI511685	*A.caudatus*	Peru	White
10	PI511687	*A.caudatus*	Peru	White
11	PI511688	*A.caudatus*	Peru	White/Brown
12	PI511689	*A.caudatus*	Peru	White/Brown
13	PI511691	*A.caudatus*	Peru	White/Brown
14	PI511693	*A.caudatus*	Peru	White
15	PI511698	*A.caudatus*	Peru	White
16	PI511700	*A.caudatus*	Peru	Light Tan
17	PI511701	*A.caudatus*	Peru	Light Tan
18	PI511703	*A.caudatus*	Peru	White
19	PI511704	*A.caudatus*	Peru	White
20	PI553073	*A.caudatus*	USA	Pink
21	PI608019	*A.caudatus*	Ecuador	Black
22	PI642736	*A.caudatus*	USA	Pink
23	PI669838	*A.caudatus*	Bolivia	Dark Brown/White
24	PI490454	*A.quitensis*	Peru	Dark Brown
25	PI490466	*A.quitensis*	Peru	Dark Brown
26	PI490708	*A.quitensis*	Ecuador	Dark Brown
27	PI511736	*A.quitensis*	Bolivia	Black
28	PI511743	*A.quitensis*	Ecuador	Black
29	PI511745	*A.quitensis*	Ecuador	Brown
30	PI511747	*A.quitensis*	Ecuador	Brown
31	PI511751	*A.quitensis*	Peru	Brown
32	PI568154	*A.quitensis*	Bolivia	Brown
33	PI649246	*A.quitensis*	Peru	Black
34	PI652419	*A.quitensis*	Brazil	Black
35	PI669830	*A.quitensis*	Ecuador	Dark Brown
36	PI669836	*A.quitensis*	Argentina	Black
37	PI669839	*A.quitensis*	Peru	Dark Brown
38	PI288278	*A.cruentus*	India	Dark Brown
39	PI433228	*A.cruentus*	Guatemala	White
40	PI462371	*A.cruentus*	Sudan	White
41	PI482051	*A.cruentus*	Zimbabwe	Black
42	PI490662	*A.cruentus*	Benin	Dark Brown
43	PI494777	*A.cruentus*	Zambia	Brown
44	PI500267	*A.cruentus*	Zambia	Dark Brown
45	PI511715	*A.cruentus*	Guatemala	Black
46	PI511717	*A.cruentus*	Guatemala	Black

47	PI511718	*A.cruentus*	Guatemala	Black
48	PI527570	*A.cruentus*	Rwanda	Dark Brown
49	PI536437	*A.cruentus*	Maldives	Dark Brown
50	PI649506	*A.cruentus*	Mexico	White
51	PI649515	*A.cruentus*	Mexico	White
52	PI649517	*A.cruentus*	Mexico	White
53	PI477914	*A.cruentus*	Mexico	Red
54	PI647848	*A.cruentus*	Russian	White
55	Ames31991	*A.cruentus*	USA	White
56	PI451711	*A.cruentus*	Mexico	Dark Brown
57	PI511726	*A.cruentus*	Mexico	White/Gold/Brown
58	PI511727	*A.cruentus*	Mexico	White
59	PI511876	*A.cruentus*	Mexico	White
60	PI538320	*A.cruentus*	USA	White
61	PI566896	*A.cruentus*	USA	Black
62	PI604556	*A.cruentus*	Mexico	White
63	PI511719	*A.cruentus*	Guatemala	White
64	PI576477	*A.cruentus*	Nigeria	Dark Brown
65	PI605353	*A.cruentus*	China	Black
66	PI674256	*A.hypochondriacus*	India	White
67	PI674257	*A.hypochondriacus*	India	White
68	PI210995	*A.hypochondriacus*	Afghanistan	Dark Brown
69	PI274279	*A.hypochondriacus*	India	White
70	PI337611	*A.hypochondriacus*	Uganda	White
71	PI477915	*A.hypochondriacus*	India	White
72	PI477916	*A.hypochondriacus*	Mexico	White
73	PI477917	*A.hypochondriacus*	Mexico	Gold
74	PI490758	*A.hypochondriacus*	Mexico	White
75	PI511721	*A.hypochondriacus*	Mexico	Tan/Dark Brown
76	PI511731	*A.hypochondriacus*	Mexico	White
77	PI511877	*A.hypochondriacus*	Mexico	White
78	PI558499	*A.hypochondriacus*	USA	White
79	PI666334	*A.hypochondriacus*	USA	White
80	PI480592	*A.hypochondriacus*	India	White
81	PI480608	*A.hypochondriacus*	India	Brown
82	PI480711	*A.hypochondriacus*	India	Dark Brown/White
83	PI480755	*A.hypochondriacus*	India	Dark Brown/White
84	PI480918	*A.hypochondriacus*	India	Brown
85	PI481023	*A.hypochondriacus*	India	Black
86	PI481126	*A.hypochondriacus*	India	Brown
87	PI481134	*A.hypochondriacus*	India	Dark Brown/White
88	PI490752	*A.hypochondriacus*	Guatemala	Brown/Dark Brown/White
89	PI540446	*A.hypochondriacus*	Pakistan	Dark Brown
90	PI572259	*A.powellii subsp. powelli*	Slovakia	Brown
91	PI572260	*A.powellii subsp. powelli*	France	Brown
92	PI604671	*A.powellii subsp. powelli*	USA	Brown
93	PI636366	*A.retroflexus*	Unknown	Black
94	PI649310	*A.retroflexus*	Mongolia	Black
95	PI633586	*A.palmeri*	Africa	Black
